# Determinants of secondhand clothing consumption in Hong Kong: An extended Knowledge-Attitude-Practice (KAP) model

**DOI:** 10.1371/journal.pone.0356518

**Published:** 2026-09-21

**Authors:** Sai-Leung Ng

**Affiliations:** Department of Geography, Chinese Culture University, Taipei, Taiwan; International Islamic University Malaysia, MALAYSIA

## Abstract

As secondhand clothing gains popularity, its environmental and sustainable consumption implications have attracted growing attention. Various behavioral models have been used to explain and predict secondhand clothing purchases. However, many studies overlook contextual factors that directly influence secondhand purchase and mediate the impact of psychological factors on consumer behavior. This study extends the Knowledge-Attitude-Practice (KAP) model by incorporating two contextual factors to examine the structural relationships among knowledge, attitude, price consciousness, perceived risk, and secondhand clothing purchase behavior. Based on an online survey of 545 respondents in Hong Kong, the study employs structural equation modeling (SEM) to analyze these relationships. Results indicate that environmental knowledge and perceived risk directly influence secondhand clothing purchase behavior. Although attitude does not directly affect behavior, it exerts an indirect effect through perceived risk. Price consciousness, however, neither affects nor mediates purchasing decisions. These findings suggest that secondhand clothing purchase in Hong Kong is driven more by environmental knowledge and risk perceptions than by economic considerations. The negligible role of price consciousness may reflect Hong Kong’s highly competitive duty-free retail environment, where the price advantage of secondhand clothing is less pronounced. This study contributes to the literature by demonstrating how contextual market conditions shape the relative importance of psychological and economic factors in sustainable consumption and offers practical recommendations for promoting secondhand clothing adoption in Hong Kong.

## 1. Introduction

Clothing is a vital element of human existence and well-being. While it serves practical functions such as keeping warm, protection from the elements and modesty, its importance extends beyond mere utility. Clothing plays a significant role in fulfilling psychological and social needs, including self-expression, cultural identity, and social integration [1]. In many societies, having access to suitable clothing is crucial for engaging in various facets of daily life, such as employment, entertainment, education, and social interactions. Additionally, clothing can impact individuals’ sense of dignity and confidence.

Population growth and modernization have led to substantial expansion in the global fashion market, reaching an annual revenue of USD 1.5 trillion [2]. However, the current methods of designing, producing, and using clothing are inefficient and harmful to the environment. It is estimated that textile production alone contributes approximately 1.2 billion tonnes of greenhouse gas emissions each year, surpassing the emissions from all international flights and maritime shipping combined [3]. Additionally, textile production is intrinsically wasteful, with more than 35% of materials in the apparel supply chain becoming waste even before a garment is purchased by consumers [4].

The materialistic consumerism lifestyle has resulted in clothes being materially under-utilized, which has proved unsustainable. The average frequency with which a piece of clothing is worn before being discarded fell by 36% from 2000 to 2015 [3]. The accumulation of waste shows no indication of decreasing, with an additional 57 million tonnes of fashion waste anticipated to be produced each year (Global Fashion Agenda, 2017). Approximately three-quarters of clothing materials ultimately end up being incinerated or sent to landfills [5]. Currently, a very small fraction (i.e., about 1%) of clothing material is recycled into new garments [2]. The under-utilization of clothing and the lack of recycling contribute to an annual economic loss exceeding USD 500 billion [3].

The circular economy represents a potential solution to the unsustainable use of clothing [5]. The circular economy aims to preserve resources such as raw materials, energy, and water, as well as reduce wastes, while also lowering carbon emissions and environmental impacts [6]. Unlike the traditional economy model, which discards the materials as waste after use, the circular economy encourages reuse and recycling, which help ensure that products or materials continue to be utilized while allowing natural systems to regenerate through biological and technical processes [5]. In this sense, extending clothing lifespans through reuse can contribute to sustainability transitions by reducing demand for new production and limiting downstream waste.

Recently, there has been a movement away from consumerism and materialism towards more responsible consumption practices [4]. The concerns about sustainability have begun to increase consumers’ interest in the environmental implications of buying clothing [7,8]. Buying secondhand clothing is consistent with the United Nations Sustainable Development Goals (SDGs), specifically with regard to “Responsible Consumption and Production” [9]. As a demand-side practice, secondhand consumption can support waste prevention and resource conservation, which are central to sustainable development pathways.

The popularity of secondhand clothing has increased in the recent decade [10,11]. Approximately one out of five American shoppers regularly visit thrift stores [12]. Over 15% of Americans shop at a thrift store at least once yearly [13]. Secondhand clothing has created a set of niche markets, with young consumers and those with limited budgets as these markets’ major targets [14]. Globally, the secondhand clothing industry has grown more than 10% per year; the revenue reached over USD 30 billion in the 2020s [15].

While the purchase of secondhand clothing has emerged as a rising global trend, researchers, particularly in consumer and retail studies, are keen to explore the factors that influence purchasing behaviors [14,16]. Viewed as a pro-environmental behavior, various behavioral models have been utilized to gain insights into the psychological factors that either encourage or discourage the buying of secondhand clothing [17,18]. One strength of behavioral models is their systematic approach, which breaks down behavior into distinct components, facilitating targeted interventions [19]. By identifying specific determinants or predictors of behavior, these models can inform the development of tailored strategies for behavior change. Additionally, behavioral models provide a theoretical basis for empirical research, allowing for testing hypotheses and evaluating interventions.

Because most behavioral models are designed for understanding general human behaviors, they tend to neglect the influences of contextual factors when making the decision to take action. Moreover, behavioral models often posit direct and linear causal relationships between psychological factors and behaviors, potentially overlooking the complexity of consumption decisions in real-world contexts. While previous studies have established the KAP framework as a useful approach for explaining sustainable consumption behavior, less is known about how economic and cultural barriers mediate the relationships among knowledge, attitude, and secondhand clothing purchase, particularly in high-density urban Asian contexts such as Hong Kong.

Secondhand consumption behavior should be examined through both economic and cultural perspectives [20]. Economic and cultural factors not only directly influence secondhand purchase but also mediate the impact of psychological factors on consumer behaviors [14]. Accordingly, this study incorporates price consciousness as an economic factor and perceived risk as a cultural factor to extend the Knowledge-Attitude-Practice (KAP) model and examine their mediating roles in secondhand clothing purchase behavior. Data was collected from an online survey (n = 545) in 2021 and, subsequently, analyzed the relationships between knowledge, attitude, price consciousness, perceived risk, and secondhand clothing purchase using structural equation modeling (SEM). The findings of this study contribute to the existing literature by providing a novel understanding of sustainable fashion consumption behavior and can help inform strategies for promoting sustainable consumption practices in Hong Kong.

## 2. Literature review

### 2.1. Secondhand clothing

Secondhand purchase refers to the purchase of previously owned or used items through alternate channels to the conventional retail market for new products [21]. Clothing and fashion items are among the most popular items sold at secondhand stores [12]. Secondhand clothing differs conceptually and operationally from vintage fashion, though they are sometimes used interchangeably by some authors [22]. Secondhand fashion refers to gently used clothing that is often sold at a discount. In contrast, vintage fashion is rare and authentic in relation to a specific time period or fashion style [23]. Some vintage fashion items may have been lightly used, but others could be new [24].

Purchasing secondhand products has become a growing trend in recent decades [15,25]. From a supply standpoint, the current success of the secondhand clothing industry is closely related to changes in disposal habits among consumers. Instead of disposing of clothing as garbage, more people are willing to donate to thrift stores, give it to family and friends, repurpose, rejuvenate, recycle, or sell it through various channels [26]. These disposal pathways support reuse and waste prevention, which are central principles in circular economy approaches to textiles.

From a demand standpoint, increasing environmental awareness and interest in sustainability have globally impacted consumers’ attitudes, behavior, and perception towards secondhand clothing [15,27]. Furthermore, the low cost of secondhand clothing appeals to a wide range of consumers [17]. Although secondhand clothing retailing was often portrayed as an informal business with low quality, thus reducing its perceived value to consumers, the stigma of purchasing secondhand clothing is fading [10]. Adding all these favorable factors together, secondhand clothing purchase is no longer limited to specific consumer groups.

The number of secondhand stores or consignment shops has grown at about ten times the rate of other stores [14]. Take two non-profit thrift stores, Goodwill and Savers, in the United States as examples; the former now is operating over 2500 stores, and the latter is running 270 shops, respectively. The number of stores increased by approximately 7% annually [13]. Alongside the growth of thrift shops, various secondhand clothing trading channels, such as flea markets, consignment stores, and online platforms, etc., have emerged [15]. Consumers’ different needs, motivations, and purchasing behaviors are evident in their selection of secondhand channels [28]. From a sustainability perspective, these channels can be understood as enabling infrastructures that extend garment lifespans and reduce textile waste.

### 2.2. Knowledge-Attitude-Practice (KAP) model

A number of behavioral models have been proposed to understand and explain the determinants of behavior. Among these models, the KAP (also called Knowledge-Attitude-Behavior, KAB) model is one of the most popular models because it deals with three fundamental constructs, namely knowledge, attitude, and behavior [29].

The KAP model was first introduced to study family planning in the 1950s [30]. Nowadays, it has been widely used to examine pro-environmental behaviors across different contexts, including green consumption [31]. As a simple and policy-relevant framework, KAP has also been used to inform interventions that promote responsible consumption, which is closely aligned with sustainable development objectives.

The basic premise of the KAP model is that knowledge affects attitude, and together, they serve as essential foundations for shaping behavior [32]. Knowledge is considered the cognitive and rational understanding of a studied object, attitude represents the affective response to that object, and behavior reflects the way that people demonstrate their knowledge and attitude through taking actions s [33].

Environmental knowledge refers to the comprehension of concepts, facts, and information related to the environment [34]. Knowledge can be further differentiated into declarative knowledge (i.e., knowing “what”) and procedural knowledge (i.e., knowing “how”) [35]. Previous studies indicated individuals’ understanding of an environmental problem positively correlates with environmental behavior [36,37]. When people have sufficient knowledge of how to protect the environment, they may be motivated to take action [38]. Strong environmental knowledge has been found to positively influence the green purchasing behavior of young consumers in Hong Kong [39]. Conversely, a lack of environmental knowledge negatively impacts green purchasing behavior [40].

Attitude has been a key research focus as it is a crucial attribute in the analysis of different environmental and consumer behaviors [41]. Environmental attitude is defined as the subjective tendency towards the value of environmental protection [42]. It refers to the combination of beliefs and concern about certain issues, people, or objects directly relevant to the environment [37]. Attitude is the predisposition of environmental behavior, as people actively strive to reduce the negative effects of their actions on the environment [43]. Environmental attitude is commonly perceived as the precondition for achieving environmental behavior [42]. Consumers’ attitude toward the environment significantly influences their purchasing decisions regarding green products [44]. Environmental attitude has been identified as a key factor in the decision to buy organic and eco-friendly food [45]. Attitudes toward air pollution have also been found to play a crucial role in shaping consumer behavior when selecting gasoline brands [46]. Likewise, environmental attitude was found to correlate with secondhand clothing purchase [12,47]**.** Furthermore, environmental knowledge was significantly correlated with environmental attitude [37]**.** A positive effect of environmental knowledge on attitude has also been reported, although the effect was not strong [48].

Overall, previous studies suggest that environmental knowledge and environmental attitude are important antecedents of pro-environmental behavior, although the strength of these relationships may vary across contexts, and positive environmental attitudes do not always translate into actual behavior. Nevertheless, the KAP model provides a useful foundation for understanding sustainable consumption. Three hypotheses are formulated:

H1: Environmental knowledge directly affects secondhand clothing purchase.
H2: Environmental attitude directly affects secondhand clothing purchase.
H3: Environmental knowledge directly affects environmental attitude.

### 2.3. Contextual mediators

While environmental knowledge and attitude are commonly regarded as key determinants of pro-environmental behavior, empirical findings remain inconsistent. Although many studies have reported positive effects, others have found moderate, weak, or indirect relationships between psychological factors and actual behavior. These inconsistencies suggest that the translation of environmental knowledge and attitudes into behavior may depend on contextual conditions. The attitude-behavior gap has been highlighted in the purchase of sustainable fashion products [49]. Although consumers often express environmental concern, they may face challenges in adopting sustainable consumption practices [50]. Similarly, a local study also reported that, even though Hong Kong consumers have a positive environmental attitude and are aware of the importance of sustainable consumption, relatively few people purchase sustainable products [51].

The existing literature has often neglected contextual variables when knowledge and attitude are used to explain consumer behaviors [52]. Contextual variables encompass environmental, social, cultural, and situational factors that influence individual behavior outcomes in a specific context [53]. Knowledge is often insufficient for taking action, although it is necessary [54]. Knowledge and attitude may materialize as behavior if contextual conditions are favorable.

A number of contextual variables affecting consumer behaviors, e.g., lifestyles [55], uncertainties [56], financial constraints [57], prior experience [58], and convenience [59], etc., have been examined. Depending upon the specific context, contextual variables may directly affect behavior, or they mediate and/or moderate the effects of psychological factors on behavior [60].

In the context of secondhand consumption, the importance of economic and cultural factors in influencing purchasing behavior has been highlighted [20]. Following this argument, the effect of price consciousness on secondhand clothing purchase has been investigated [61], while the influence of cultural perceptions of vintage items on secondhand purchasing behavior has also been examined [10].

Price consciousness (or price sensitivity) refers to the extent to which individuals are concerned with paying a low price [62,63]. Price consciousness has been identified as a primary motivator for consumers to purchase secondhand products [28,61,64]. It has also been reported as a driver of secondhand purchasing behavior [14]. Consumers who are high in price consciousness will display lower demand as price increase, whilst consumers low in price consciousness will not react as strongly to price differences [14]. Compared to brand-new clothing, the bargain prices of secondhand products are especially attractive to financially disadvantaged individuals [10] and frugal consumers [14]. In this regard, this study conceptualizes price consciousness as a tendency to emphasize obtaining products at low prices and exercising frugality, rather than as an evaluation of overall product value or value for money. One hypothesis is formulated:

H4: Price consciousness affects secondhand clothing purchase.

Knowledge and attitude toward environmental sustainability can influence the importance consumers attach to price when making purchasing decisions. Consumers with stronger environmental knowledge and more positive environmental attitudes may place greater value on the environmental benefits of secondhand clothing and therefore rely less on price-based evaluations [65]. In this context, price consciousness refers to the tendency to focus on obtaining products at the lowest possible price (Palazón & Delgado, 2009). Individuals who prioritize environmental outcomes may be less sensitive to price differences because the environmental value of secondhand clothing forms part of the product’s perceived value [66]. Consequently, environmental knowledge and attitude may reduce the degree to which consumers emphasize low prices when considering secondhand clothing purchases. Therefore, two hypotheses are formulated:

H5: Environmental knowledge affects price consciousness.
H6: Environmental attitude affects price consciousness.

On the other hand, the worry about negative or unpleasant consequences associated with secondhand clothing is referred to as perceived risk [67]. In the old days, secondhand items were perceived as a sign of poverty [68]. Even in the present, some people may still perceive secondhand clothing as “old and tatty,” “out of fashion,” and “not the best quality” [18]. In the Western world, the stigma surrounding purchasing secondhand clothes is diminishing, as it’s no longer confined to specific societal segments, as was customary in the past [10]. However, consumers’ intentions to buy secondhand products are frequently influenced by concerns about potential contamination from previous usage [28]. Despite awareness of the environmental and social benefits of secondhand clothing, many consumers remain reluctant to purchase such products [68]. In oriental cultures, particularly Chinese culture, there’s a general aversion to wearing secondhand clothing. Influenced by traditional beliefs, concerns arise about the transfer of misfortune and the negative impact on one’s public image, potentially suggesting financial incapacity to buy new clothing [18]. In short, consumers’ perceived risk of secondhand clothing might lead to a barrier to sustainable fashion consumption, which varies in each community. Therefore, one hypothesis is formulated:

H7: Perceived risk of secondhand clothing affects secondhand clothing purchase.

On the other hand, individuals with good knowledge and positive attitudes toward environmental sustainability may view secondhand clothing more favorably because of its alignment with sustainable principles [69]. These individuals may perceive secondhand items as environmentally friendly, ethical, and supportive of reducing waste and consumption. Their positive attitudes toward sustainability may shape their perceptions of secondhand items as desirable, acceptable, and even fashionable choices [10]. Therefore, two hypotheses are formulated:

H8: Knowledge affects perceived risk of secondhand clothing.
H9: Attitude affects perceived risk of secondhand clothing.

**Fig 1:**
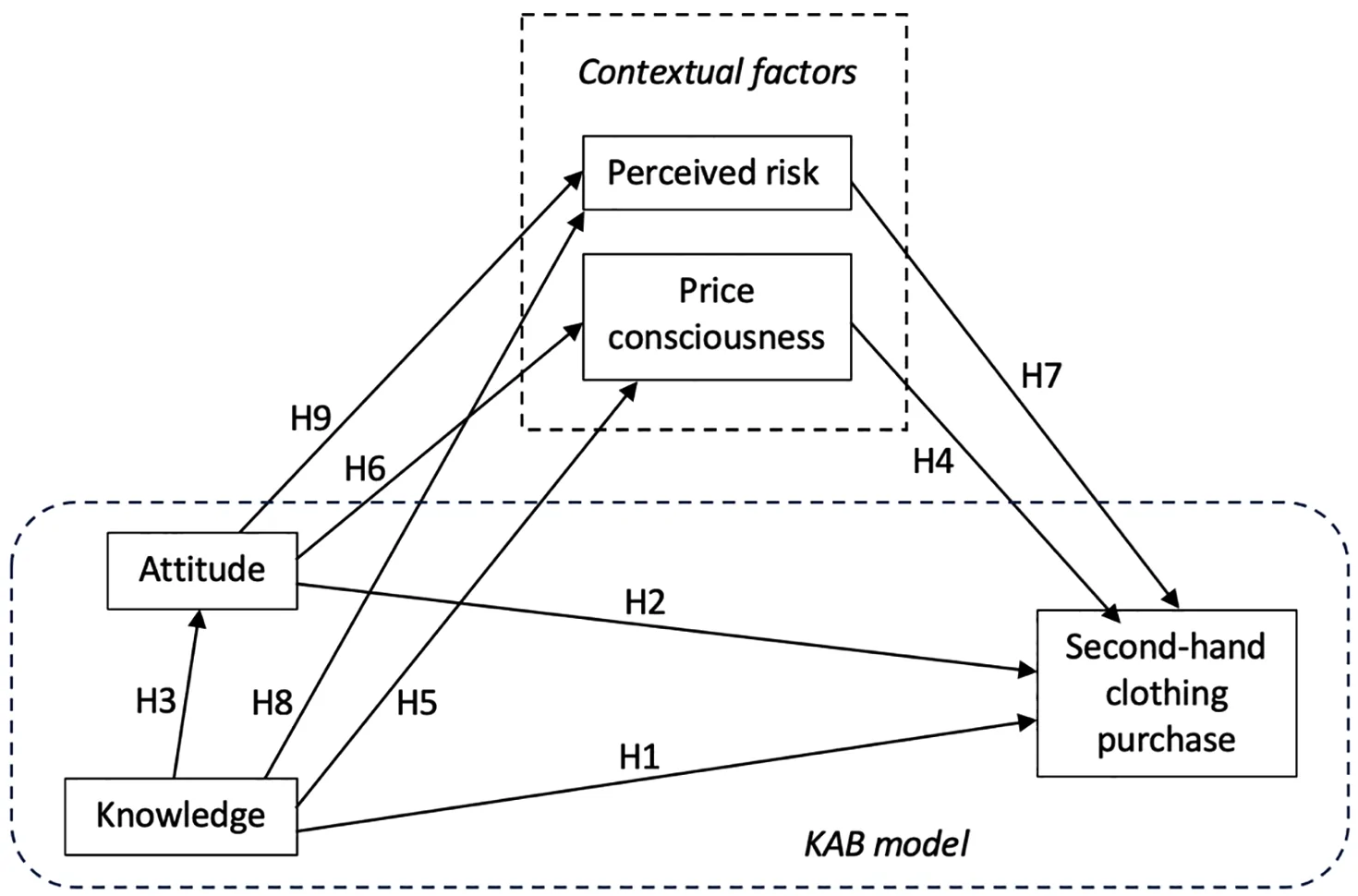
Conceptual framework of this study.

By integrating the above hypotheses, this study proposes an extended KAP model with the addition of two contextual variables as mediators (Fig 1). It is hoped that the extended KAP model can help clarify the complex relationships between knowledge, attitude, behavior, price consciousness and perceived risk. On the one hand, both psychological factors (i.e., knowledge and attitude) and contextual factors (i.e., price consciousness and perceived risk) may have direct effects on secondhand clothing purchase. On the other hand, psychological factors may generate indirect (i.e., mediation) effects on secondhand clothing purchase through contextual factors (i.e., mediators). This integrated perspective is consistent with the view that responsible consumption is shaped by both individual dispositions and the enabling or constraining conditions of the consumption environment.

## 3. Methods

### 3.1. Measurement instrument

The questionnaire was developed based on a conceptual framework that comprised five constructs, namely knowledge, attitude, practice, price consciousness, and perceived risk of secondhand clothing (Table 1). To ensure the validity of the survey, question items were adapted from previous relevant studies and revised to fit the context of this study.

**Table 1:** Constructs and question items of this study.

Construct	Item and Description		Reference
Knowledge	KW1	I know the meaning of sustainable consumption	[31,32,44,60,69,70]
	KW2	I know the definition of sustainable fashion	
	KW3	I know how to do sustainable consumption	
	KW4	I don’t know where I can buy sustainable fashion	
Attitude	AT1	I feel that Hong Kong needs to promote sustainable consumption	
	AT2	I feel that sustainable consumption cannot reduce the problem of urban waste	
	AT3	I think that everyone has a responsibility to protect the environment	
	AT4	I think that it is important to raise the environmental awareness of consumers in Hong Kong	
Behavior	BH	The frequency of purchasing secondhand clothing	
Price consciousness	PS1	Price is the most important consideration factor when I purchase fashion products.	[21,28,62,64,71]
	PS2	Before purchasing a product, I will compare prices first	
	PS3	The price of sustainable fashion is generally acceptable	
Perceived risk	SCP1	I think wearing second-hand clothing will affect my image	[14,18,28,63,67]
	SCP2	I think wearing second-hand clothing will affect my luck	
	SCP3	I think second-hand clothing is often perceived as having a poor and worn-out reputation	

The questions for environmental knowledge included a few statements regarding their declarative knowledge and procedural knowledge about sustainable clothing [31,32,44,60,69,70]. The section on environmental attitude included several statements related to belief, cognition, and concern towards sustainable clothing and environmental sustainability [31,32,44,60,69,70]. Price consciousness was measured by asking respondents’ agreement with price importance and frugality [21,62,64,68,71]. Perceived risk of secondhand clothing was assessed by respondents’ perceived values and risks of secondhand clothing, including the personal and social consequences [14,18,28,63,67]. Referring to the practice of secondhand clothing purchase, respondents were asked to indicate how frequently they purchase secondhand clothing. Lastly, the information about respondents’ gender, age, education level, and monthly income was collected to understand the demographic profile of the sample.

Respondents were asked to indicate their agreement with each latent variable using a five-point Likert scale, with 1 denoting “strongly disagree” and 5 denoting “strongly agree.” This technique is commonly used in social and psychological studies to collect scaled data on opinions. Two independent experts in the field reviewed the questionnaire to improve the face validity. A pilot survey was conducted to refine any unclear or ambiguous terminology. These procedures were intended to support the clarity and validity of the instrument in the study context.

### 3.2. Sampling

Although secondhand clothing is popular in certain social classes or cohorts, it has not yet become mainstream in society. Therefore, appropriate respondents may be difficult to reach through traditional sampling methods. Accordingly, this study conducted a web-based questionnaire survey to improve data collection efforts [72].

Ethical approval for this study was obtained from the Research Administration Office of The Chinese University of Hong Kong. An introductory statement was presented at the beginning of the online questionnaire, informing potential respondents about the purpose of the study, the voluntary nature of participation, and the assurance of anonymity and confidentiality. Consent to participate was implied by respondents’ decision to proceed with the survey.

A snowball strategy was employed to recruit respondents who had purchased or were interested in secondhand clothing [73]. After completing the questionnaire, the respondents were invited to forward the questionnaire to other potential respondents to generate the snowball effect. Web-based snowball sampling surveys have proven effective in investigating green consumption [44].

Table 2 shows the demographic profile of the respondents. The number of females (60.8%) was larger than males (39.2%). The distribution of age groups revealed that around half of the respondents (50.2%) belonged to the 18−22 age group, with the remaining groups categorized as under 18, 23−29, 30−39, 40−49, and 50−59, constituting 19.4%, 13.4%, 8.4%, 6.7%, and 1.7%, respectively. The over-representation of young consumers aligns with previous studies, indicating that youths, particularly students, are actively engaged in the purchase of secondhand clothing [14]. Regarding educational attainment, approximately half of the respondents had an education level of college or higher (48.3%). In comparison, 37.1% had completed secondary education, possibly influenced by the 28.6% of respondents under the age of 18. Additionally, 12.7% had post-secondary education qualifications such as a higher diploma or associate degree. In relation to income levels, 55.8% reported earning less than HKD 10,000 per month, a trend likely attributed to a significant portion of students among the respondents. Individuals earning between HKD 10,000-$19,999 monthly constituted approximately 30.1% of the sample, while 11.7% reported earning HKD 20,000−29,999 monthly. Further, respondents with monthly incomes ranging from HKD 30,000−39,999 and HKD 40000−49,999 represented 1.5% and 0.9% of the sample, respectively.

**Table 2:** Demographic profile of the respondents.

Characteristic	Category	Number (%)
Gender	1. Male	214 (39.2%)
	2. Female	331 (60.8%)
Age	1. < 18	106 (19.4%)
	2. 18-22	274 (50.2%)
	3. 23-29	73 (13.4%)
	4. 30-39	46 (8.4%)
	5. 40-49	37 (6.7%)
	6. 50-59	9 (1.7%)
	7. > 60	0 (0%)
Education Level	1. Primary or below	0 (0%)
	2. Secondary	202 (37.1%)
	3. Post-secondary diploma or Certificate	69 (12.7%)
	4. College, university, or above	263 (48.3%)
	5. Refuse to disclose	12 (2.2%)
Monthly income (US$1 = HK$7.8)	1. < HK$10,000	304 (55.8%)
	2. HK$10,000–19,999	165 (30.1%)
	3. HK$20,000–29,999	63 (11.7%)
	4. HK$30,000–59,999	8 (1.5%)
	5. HK$40,000 or above	5 (0.9%)

### 3.3. Data analysis

All questionnaire responses were reviewed for completeness, with incomplete or unresponsive entries removed. Eventually, 545 valid cases were retained for statistical analysis. The sample size was deemed sufficient for conducting structural equation modeling [74].

Descriptive statistics were utilized to classify respondents into demographic groups. The numerical data for the five constructs were assessed for normality, with skewed data undergoing log transformation. Cronbach’s alpha values were used to assess the reliability of latent variables. The common latent factor accounted for 36.4% of the total variance, which was below the recommended threshold of 50%, suggesting that common method bias was unlikely to be a serious concern. These statistical analyses were conducted using IBM SPSS Statistics 26.0.

SEM was performed to investigate the relationships among five constructs in the conceptual framework using AMOS ver. 26.0. SEM is a powerful statistical technique that allows for the examination of complex relationships among latent (unobserved) variables and observed variables simultaneously (Kline, 2010). This method consists of two main steps, namely validating the measurement model and testing the structural model [75].

The measurement model defines the relationships between latent variables and their corresponding observed items. To assess its validity, confirmatory factor analysis was conducted, examining the connections between latent constructs and their observed indicators. The validity and overall fit of the measurement model were confirmed, as all required indices met the recommended thresholds (Tables 3 and 4).

**Table 3:** Assessment results of the measurement model.

Construct	Cronbach’s alpha	Construct reliability	Average variance extracted	Construct			
				Knowledge	Attitude	Price consciousness	Perception risk
Knowledge	0.880	0.883	0.716	0.846^a^	--	--	--
Attitude	0.884	0.852	0.590	0.307	0.768^a^	--	--
Price consciousness	0.965	0.975	0.952	0.088	0.025	0.976^a^	--
Perception risk	0.764	0.782	0.547	0.095	0.069	0.070	0.740^a^
Recommended threshold	>0.7	>0.6	>0.5	All the correlations between constructs were lower than square roots of the values of average variances extracted			

(^a^ The square root of the average variance extracted of the construct).

**Table 4:** Indexes of fit of the measurement and structural models.

Fit index	Accepted value	This study	
		Measurement model	Structural model
Normed Chi-square (χ²/df)	<5	1.673	1.643
Root Mean Square Error of Approximation (RMSEA)	<0.08	0.035	0.034
Goodness-of-Fit Index (GFI)	> 0.8	0.976	0.974
Comparative fit index (CFI)	>0.9	0.991	0.990
Normed Fit Index (NFI)	>0.9	0.977	0.974
Relative Fit Index (RFI)	>0.9	0.969	0.964
Incremental Fit Index (IFI)	>0.9	0.991	0.990
Tucker-Lewis Index (TLI)	>0.9	0.987	0.986

Subsequently, the structural model was developed to analyze the causal relationships between latent variables. The model demonstrates a good fit to the data, as all fitness criteria are satisfied (Table 4). The structural model defines the directional relationships between latent variables based on the conceptual framework. Maximum likelihood estimation was applied to evaluate the significance and strength of these relationships. Additionally, the indirect effects of mediating variables were evaluated using 95% confidence intervals derived from 2,000 bootstrap samples.

## 4. Results

### 4.1. Univariate statistics

Table 5 shows the respondents’ levels of knowledge, attitude, price consciousness, perceived risk, and secondhand clothing purchase. The respondents had a moderately low level of environmental knowledge (2.897 ± 0.821), yet they held a notably positive attitude toward environmental issues (4.253 ± 0.638). Additionally, respondents showed a moderate sensitivity to price (3.473 ± 0.731) but displayed a relatively low perceived risk of secondhand clothing (1.905 ± 0.790) and engaged in a low level of secondhand clothing purchase (1.950 ± 0.925). In general, demographic variables did not well predict respondents’ psychology and behavior, except that knowledge and attitude were significantly affected by age (Table 5). Overall, these results suggest that respondents expressed strong pro-environmental attitudes despite comparatively lower environmental knowledge and low levels of secondhand purchase.

**Table 5:** Results of ANOVA testing the effects of demographics on knowledge, attitude, price consciousness, perception concern, and secondhand clothing purchase.

Construct	Mean Std. dev.	F-value			
		Gender	Age	Education	Income
Knowledge	2.897 ± 0.821	0.747	4.746**	0.752	0.742
Attitude	4.253 ± 0.638	0.579	3.298**	0.901	0.679
Price consciousness	3.473 ± 0.731	3.535	0.999	0.279	0.311
Perception concern	1.905 ± 0.790	0.258	1.105	0.279	0.543
Secondhand clothing purchase	1.950 ± 0.925	0.304	1.683	0.996	1.064
(Note: **p < 0.01)					

### 4.2. Verification of hypotheses

**Fig 2:**
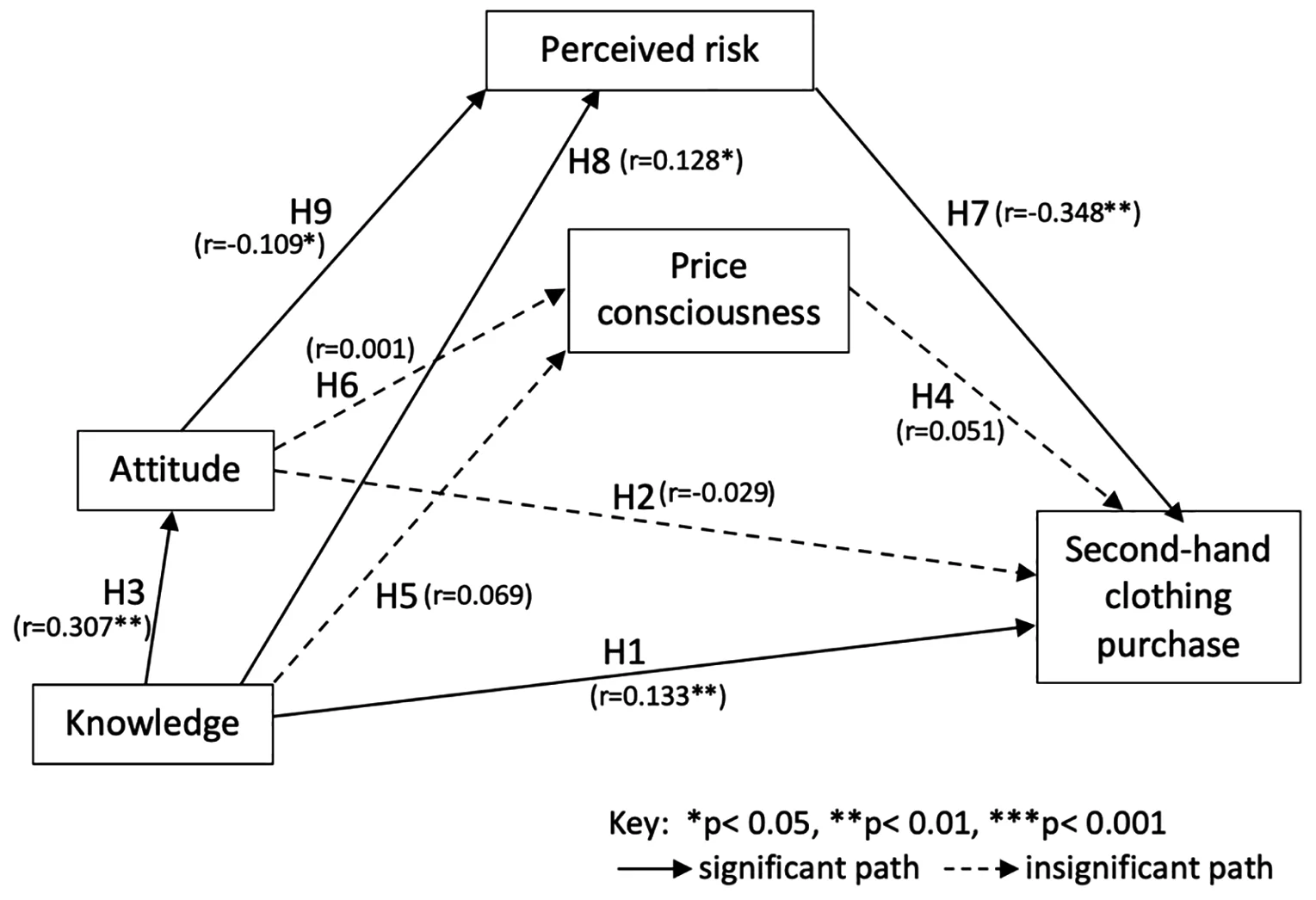
SEM of this study.

Because the validity and reliability of the measurement model had been confirmed, proceeding with the structural model analysis was appropriate. The path coefficients between latent variables were estimated for the verification of hypotheses (Fig 2). At a 0.05 significance level, H1, H3, H7, H8, and H9 were accepted but H2, H4, H5, and H6 were rejected (Table 6). Specifically, environmental knowledge had significant direct effects on environmental attitude and secondhand clothing purchase, but environmental attitude did not significantly affect secondhand clothing purchase. Perceived risk also had a significant effect on secondhand clothing purchase, while knowledge and attitude significantly influenced perceived risk. By contrast, the hypothesized paths involving price consciousness were not supported.

**Table 6:** Verification of hypotheses.

Hypothesis (Relation)	Estimate	Standard error	Standardized path coefficient	Critical ratio	Decision
H1 (Knowledge → Secondhand clothing purchase)	0.140	0.049	0.133	2.869**	Accepted
H2 (Attitude → Secondhand clothing purchase)	−0.046	0.074	−0.029	−0.622	Rejected
H3 (Knowledge → Attitude)	0.204	0.032	0.307	6.293**	Accepted
H4 (Price consciousness → Secondhand clothing purchase)	0.043	0.032	0.051	1.315	Rejected
H5 (Knowledge → Price consciousness)	0.088	0.048	0.069	1.823	Rejected
H6 (Attitude → Price consciousness)	0.002	0.066	0.001	0.031	Rejected
H7 (Perception risk → Secondhand clothing purchase)	−0.399	0.055	−0.348	−7.215**	Accepted
H8 (Knowledge → Perception risk)	0.118	0.049	0.128	2.393*	Accepted
H9 (Attitude → Perception risk)	−0.150	0.075	−0.109	−1.991*	Accepted

(Note: *p < 0.05 if Critical Ratio > 1.96, **p < 0.01 if Critical Ratio > 2.576).

## 5. Discussion

### 5.1. KAP of secondhand clothing purchase

H1 tested the direct effect of environmental knowledge on secondhand clothing purchase. Because environmental knowledge significantly correlated with purchasing behavior (r = 0.133, CR = 2.869, p < 0.01), H1 was accepted. With an adequate amount of environmental knowledge, consumers are able to know what kind of actions could help protect the environment and are more inclined to buy secondhand clothing. The finding is consistent with previous studies (e.g., Lee, 2010).

H2 tested the direct effect of environmental attitude on secondhand clothing purchase. Because environmental attitude did not significantly correlate with secondhand clothing purchase (r=−0.029, CR = −0.622, p > 0.05), H2 was rejected. This finding is not consistent with many previous studies, showing that environmental attitude had a significant effect on secondhand clothing purchase [12,47]. Nevertheless, young generations do not often materialize environmental attitudes to behavior accordingly [14,76]. In this study, the non-significant attitude-behavior relationship suggests the presence of an attitude-behavior gap in secondhand clothing purchase, even when pro-environmental attitudes are relatively high. This finding is consistent with the univariate statistics, which show that respondents reported a highly positive environmental attitude (4.253 ± 0.638) but a low level of secondhand clothing purchase (1.950 ± 0.925). The descriptive contrast between favorable attitudes and limited purchasing behavior highlights the importance of examining additional factors that may constrain the translation of environmental concern into actual consumption practices.

H3 tested the direct effect of environmental knowledge on attitude. Because environmental knowledge significantly correlated with attitude (r = 0.307, CR = 6.293, p < 0.01), H3 was accepted. This finding is consistent with previous studies, suggesting that the more knowledgeable the consumers are about the environment, the more positive they are towards the environment [37]. When consumers become more knowledgeable about environmental issues, their attitudes tend to shift in response to their newly acquired knowledge [31]. Taken together, H1 and H3 indicate that environmental knowledge contributes to secondhand purchase primarily through a direct pathway, while the knowledge–attitude association does not translate into a direct attitude–purchase relationship in this sample.

### 5.2. Contextual factors of secondhand clothing purchase

H4 and H7 tested the influences of two contextual factors, namely price consciousness and perceived risk of secondhand clothing purchase, respectively. H4 was rejected because the correlation between price consciousness and secondhand clothing purchase was not statistically significant (r = 0.051, CR = 1.315, p > 0.05). The insignificant effect of price consciousness on secondhand purchase is probably because the brand-new merchandise is sold at a very competitive price in Hong Kong because Hong Kong is a free port without levying a customs tariff. As a result, the price difference between brand-new and secondhand clothing may not be large enough to affect secondhand purchase among respondents. This finding suggests that economic appeal may not be the primary driver of secondhand clothing purchase in this context.

On the other hand, perceived risk significantly correlated with secondhand purchase (r = −0.348, CR = −7.215, p < 0.01). Therefore, H7 was accepted. The finding is consistent with previous studies, indicating that people with a higher perceived risk of secondhand clothing were less likely to purchase secondhand clothing [18,28]. Perceived risk may therefore operate as a key barrier that constrains responsible consumption practices in the clothing sector. In Hong Kong, such perceptions may be reinforced by broader social and cultural concerns associated with secondhand goods. Previous studies have suggested that Asian consumers may attach symbolic meanings to previously owned possessions, which can contribute to negative perceptions of secondhand products [10]. These concerns may further discourage secondhand clothing purchase despite the environmental benefits associated with reuse.

H5 and H6 tested the influences of knowledge and attitude on price consciousness, respectively. Both hypotheses were rejected because price consciousness did not significantly correlate with knowledge (r = 0.069, CR = 1.823, p > 0.05) and attitude (r = 0.001, CR = 0.031, p > 0.05). This finding implies that environmental benefits may be more salient than price considerations among respondents, and that price differences between brand-new and secondhand clothing are not a primary determinant of purchase behavior in this sample.

H8 and H9 tested the influences of knowledge and attitude on perceived risk, respectively. Because perceived risk was significantly influenced by both knowledge (r = 0.118, CR = 2.393, p < 0.05) and attitude (r = −0.109, CR = −1.191, p < 0.05), H8 and H9 were accepted. This finding is consistent with previous studies, suggesting that consumers with positive environmental knowledge and attitude may prioritize the environmental benefit of buying secondhand over finding a flawless garment [10,69]. In other words, psychological factors may reduce perceived risk, which in turn supports secondhand purchase.

### 5.3. Contextual factors as mediators

Although the correlations indicate the direct effects of psychological and contextual factors on secondhand clothing purchase, they are not sufficient to explain why people purchase secondhand clothing. The indirect effects of knowledge and attitude on secondhand clothing purchase through mediators should also be investigated.

Perceived risk served as a mediator in the relationship between attitude and secondhand clothing while price consciousness does not. Table 7 summarizes the statistics of direct and indirect effects of knowledge and attitude on secondhand clothing purchase via perceived risk. Environmental knowledge has a significant direct effect (standardized direct effect = 0.133, p < 0.01) but an insignificant indirect effect (standardized indirect effect = −0.038, p > 0.05) on secondhand clothing purchase. The 95% confidence interval for the indirect effect of knowledge (lower = −0.082, upper = 0.001) includes zero, which supports the conclusion of no mediation. The relationship between them is straightforward, as no mediation is involved.

**Table 7:** Mediation effects of knowledge and attitude on secondhand clothing purchase.

Path		Independent variable	
		Knowledge	Attitude
Direct path: Independent variable → Secondhand clothing purchase	Standard effect	0.133**	-0.029
	Standard error	0.049	0.074
Indirect path: Independent variable → Perceived risk → Secondhand clothing purchase	Standard effect	-0.038	0.038*
	Standard error (2000 bootstrap samples)	0.025	0.020
	95% C.I. lower bound	-0.082	0.007
	95% C.I. upper bound	0.001	0.072
Type of mediation		No mediation	Full mediation

(Note: *p < 0.05, **p < 0.01).

Although the direct effect of attitude on secondhand clothing purchase is insignificant, it does not mean that attitude is irrelevant. The indirect effect of attitude via perceived risk (standardized indirect effect = 0.038, p < 0.05) is significant. The 95% confidence interval for the indirect effect of attitude (lower = 0.007, upper = 0.072) does not include zero, indicating a significant mediation effect (Table 7). Therefore, attitude plays a role in influencing purchasing decisions through shaping perceptions of risk, and the mediation is full. The mediating role of perceived risk provides a plausible explanation for the attitude–behavior gap observed in this study. Although respondents reported positive environmental attitudes, these attitudes did not directly translate into secondhand clothing purchase. Instead, favorable attitudes appear to encourage purchase only when they are accompanied by lower perceptions of risk associated with secondhand clothing. This interpretation is consistent with the negative association between attitude and perceived risk (H9) and the negative association between perceived risk and purchase (H7), which together imply a positive indirect effect of attitude on purchase through reduced perceived risk.

### 5.4. Theoretical Implications

This study proposes a comprehensive model to investigate secondhand clothing purchase. When the KAP model per se was adopted, it was found that environmental knowledge affects behavior but attitude does not. The finding of knowledge-behavior relationship is consistent with previous studies [39]. The absence of a significant correlation between attitude and behavior suggests the presence of an attitude-behavior gap in secondhand clothing purchase [49]. Some previous studies also reported that positive attitude did not necessarily translate well into practices [14,76].

When a comprehensive model that includes contextual factors is used, a different but interesting picture is produced that allows an in-depth interpretation. First, knowledge has a direct effect on behavior, and the effect is not mediated by contextual factors. In addition, knowledge significantly affects attitude, but attitude does not directly affect behavior, which suggests that the pathway from knowledge to behavior does not operate primarily through attitude in this context. The overall effect of knowledge on behavior, in terms of the strength of total effect, is stronger than that of attitude, indicating that cognition plays a more important role than affection in secondhand clothing purchase behavior.

Second, contextual factors, on the one hand, have direct effects on behavior; on the other hand, they mediate the indirect effects of psychological factors on behavior. Nevertheless, not all contextual factors can significantly affect behavior. In this study, perceived risk was a predictor and mediator of behavior, but price consciousness was not. Although secondhand clothing consumption is often assumed to be associated with economic motivations and the pursuit of lower prices, the insignificant effect of price consciousness suggests that economic considerations may not always be the dominant driver of secondhand clothing purchase, particularly in the Hong Kong context. Therefore, the selection of contextual factors is specific to the research topic and context. Furthermore, this study highlights the importance of examining context-specific mechanisms rather than assuming universal attitudinal or economic drivers of sustainable consumption.

### 5.5. Practical implications

As an emerging market for green products, Hong Kong has an unprecedented potential for secondhand clothing. However, consumer behavior regarding secondhand clothing in this region remains largely unexplored. The absence of market information often serves as a major barrier to the successful expansion of green products [77]. The lack of market information in Asian countries has caused difficulties in practicing segmentation by international green marketers [70]. Therefore, it is important to understand the factors affecting consumers’ purchasing decision and behavior on sustainable consumption. It is hoped that the findings of this study would serve as a reference and source of information for the promotion of used apparel in Hong Kong.

In this study, knowledge and perceived risk are two predictors of secondhand purchase. Because knowledge directly affects secondhand purchase, educational programs are always necessary to enhance environmental knowledge in society. Equally important are promotional strategies that reduce the negative image of secondhand clothing. Local retailers should be encouraged to reduce the perceived risk of secondhand clothing.

Three strategic considerations for secondhand items are reliability with regard to safety and health, adherence to technical standards, and durability and dependability [21]. Therefore, providing warranties and detailed product information, which are often lacking in the used goods market, is essential for mitigating risks associated with these products. When people realize the absence of risks associated with secondhand clothing, they become motivated to purchase it [28]. From a sustainable consumption perspective, these measures can help strengthen trust in reuse channels and support waste prevention efforts in Hong Kong.

### 5.6. Limitations and recommendations

This study has a few limitations that future research should address. First, the online survey may imply sampling limitations. Although the snowball sampling strategy was intentionally used to recruit individuals who had purchased or were interested in secondhand clothing, the resulting sample was concentrated among younger and lower-income respondents. Therefore, the findings should be interpreted as reflecting the characteristics of actual or potential secondhand clothing consumers rather than the general Hong Kong population. Future research may employ probability-based or stratified sampling approaches to examine whether the observed relationships, including the role of price consciousness and perceived risk, remain consistent across different demographic groups.

Second, the self-reporting questionnaire collecting opinions from respondents may imply social desirability bias [78] but there were no observed measures of behaviors in this study. Because people may behave differently from what they report, so their opinions may not accurately reflect the actual purchasing behavior. It is recommended that future research should include the measurement of secondhand behavior that allows verification.

Third, this study accepts and rejects hypotheses based on statistical probability theory, but statistical correlations are not always causal. Future research may use qualitative methods, such as in-depth interviews and focus groups, to identify causal relationships.

Fourth, the study focuses on a population living in Hong Kong’s specific social and cultural environment. Therefore, it is prudent to apply the study’s results directly to other countries and regions.

## 6. Conclusion

Secondhand clothing purchase, like other green consumption behaviors, is shaped by determinants on many different levels. This study collected first-hand data and adopted an extended KAP model to investigate how secondhand clothing purchase is affected by selected psychological and contextual variables in Hong Kong. This study reveals that Hong Kong consumers generally possess moderate environmental knowledge and a strong pro-environmental attitude. It is found that environmental knowledge and attitude had different roles in secondhand clothing purchase, although different (i.e., direct and indirect) effects are involved. Environmental knowledge had a significant direct effect on secondhand clothing purchase, while environmental attitude did not have a significant direct effect. The attitude-behavior gap could be explained by the mediation effects of perceived risk. Specifically, environmental attitude influenced secondhand clothing purchase indirectly through perceived risk, and this mediation was full. By contrast, environmental knowledge showed no significant indirect effect through perceived risk, which indicates no mediation.

In conclusion, this study supplements the existing literature on sustainable consumption and secondhand clothing purchase by offering new insights into the secondhand clothing purchasing behavior of Hong Kong residents. The findings are valuable for informing efforts to promote sustainable consumption, specifically, secondhand clothing purchase, in Hong Kong. In practical terms, initiatives that strengthen environmental knowledge and reduce perceived risk, for example by improving trust, hygiene assurance, and product information in secondhand channels, may help support the adoption of secondhand clothing as a form of responsible consumption.
